# 215. Shorter vs. Longer Duration of Treatment in Patients with Ceftriaxone-resistant and Ceftriaxone-susceptible *Escherichia coli* Bloodstream Infections

**DOI:** 10.1093/ofid/ofad500.288

**Published:** 2023-11-27

**Authors:** Natalie A Mackow, Lizhao Ge, Lauren Komarow, Wanying Shao, Erica Herc, Yohei Doi, Cesar A Arias, Owen Albin, Elie Saade, Loren G Miller, Jesse T Jacob, Michael J Satlin, Martin Krsak, W Charles Huskins, Sorabh Dhar, Samuel A Shelburne, Carol Hill, Kerryl E Greenwood-Quaintance, Suzannah Schmidt-Malan, Robin Patel, Vance G Fowler, Pranita Tamma, David van Duin

**Affiliations:** University of North Carolina, Chapel Hill, North Carolina; George Washington University, Rockville, Maryland; George Washington University, Rockville, Maryland; George Washington University, Rockville, Maryland; Henry Ford Hospital, Detroit, Michigan; University of Pittsburgh, Pittsburgh, PA; Houston Methodist and Weill Cornell Medical College, Houston, TX; University of Michigan Medical School, Ann Arbor, MI; Case Western Reserve University, Cleveland, Ohio; David Geffen School of Medicine at UCLA, Torrance, California; Emory University School of Medicine, Atlanta, GA; Weill Cornell Medicine, New York, NY; University of Colorado School of Medicine, CO; Mayo Clinic, Rochester, MN; Wayne State University/Detroit Medical Center, John Dingell VAMC, Detroit, Michigan; MD Anderson-University of Texas, Houston,, Texas; Duke Clinical Research Institute, Durham, North Carolina; Mayo Clinic, Rochester, MN; Mayo Clinic, Rochester, MN; Mayo Clinic, Rochester, MN; Duke University Medical Center, Durham, NC; Johns Hopkins School of Medicine, Baltimore, MD; University of North Carolina at Chapel Hill, Chapel Hill, NC

## Abstract

**Background:**

Several randomized controlled trials (RCT) have shown that short-course (∼7 days) antibiotic treatment is non-inferior to longer antibiotic courses (∼14 days) in patients with uncomplicated bloodstream infection (BSI) with mostly susceptible Gram-negative bacteria. Here, we evaluate short-course therapy in ceftriaxone-resistant *E. coli* BSI.

**Methods:**

In a prospective cohort of 300 patients with *E. coli* BSI at 14 United States hospitals between November 2020 and April 2021, each patient with ceftriaxone-R *E. coli* BSI, and the next consecutive patient with a ceftriaxone-S *E. coli* BSI was included. Patients who received 5-8 days (“short”) or 9-21 days (“long”) of antibiotics were included in this analysis. Patients who died before day 9 were excluded. Primary outcome was a Desirability of Outcome Ranking (DOOR, Table 1) based on disposition at day 30 after collection of the index blood culture. Ceftriaxone susceptibility was centrally determined using broth microdilution for all bacterial isolates.

**Figure:**
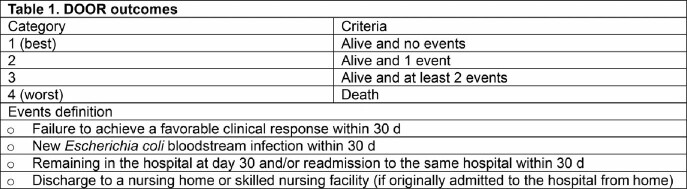

Desirability of outcome ranking (DOOR) categories

**Results:**

Of 300 patients in the original cohort, 227 were included; 44 patients (24 ceftriaxone-S, 20 ceftriaxone-R) received short (median 8 days, range 5-8 days), and 183 patients (96 ceftriaxone-S, 87 ceftriaxone-R) received long duration (median 15 days, range 9-21 days). Age (median 68 years, IQR 57-77 years), sex (125/227 [55%] female), and Charlson comorbidity index (median 2, IQR 1-4) were similar between groups. Notably, almost all patients (18/19, 95%) with solid organ or stem cell transplant were in the long duration group. The median Pitt bacteremia score was 2 (IQR 1-3) in the short duration group vs. 1 (IQR 0-3) in the long duration group (Wilcoxon Rank Sum p=0.07). DOOR outcomes were similar in both groups (Figure and Table 2). Numerically more patients in the ceftriaxone-resistant group on short treatment were in category 3; 4/20 (20%) vs 5/87 (6%) in the long duration group. These 4 patients all had unsuccessful discharge combined with renal failure (n=2), and/or lack of clinical response (n=3).

**Figure:**
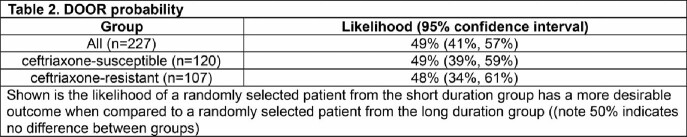

Desirability of outcome ranking (DOOR) analyses

**Figure.**
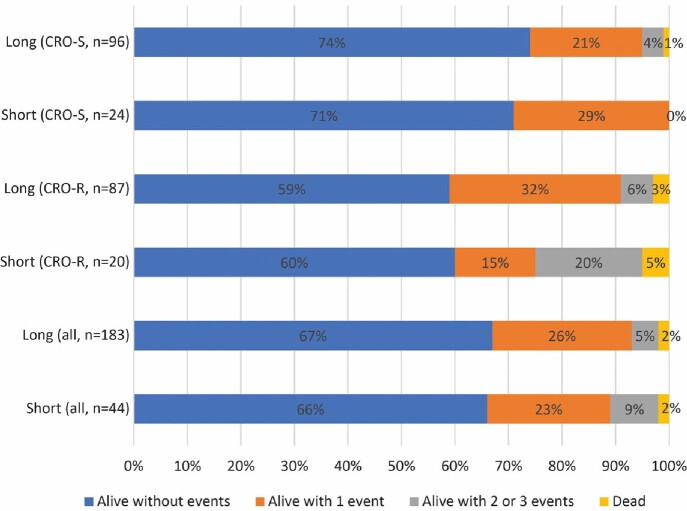
DOOR outcomes

Shown are the percentages of patients in each group with a specific DOOR category outcome.

**Conclusion:**

Short duration of therapy was less frequently used than long duration of therapy in this prospective cohort of *E. coli* BSI. Further studies are needed to determine whether short-course therapy is appropriate for ceftriaxone-R E. coli BSI.

**Disclosures:**

**Yohei Doi, MD, PHD**, bioMerieux: Advisor/Consultant|FujiFilm: Advisor/Consultant|Gilead: Advisor/Consultant|Gilead: Honoraria|GSK: Advisor/Consultant|Meiji Seika Pharma: Advisor/Consultant|Moderna: Advisor/Consultant|Moderna: Honoraria|MSD: Advisor/Consultant|MSD: Honoraria|Shionogi: Advisor/Consultant|Shionogi: Grant/Research Support|Shionogi: Honoraria **Owen Albin, MD**, Charles River Laboratories: Advisor/Consultant|Shionogi: Advisor/Consultant **Elie Saade, MD, MPH, FIDSA**, Envision Pharma: Speaker, Presenter|Johnson and Johnson: Speaker, Travel, Lodging|Protein Sciences Corp: Grant/Research Support|Sanofi Pasteur: Speaker, Travel and Lodging **Loren G. Miller, MD MPH**, ContraFect: Grant/Research Support|GSK: Grant/Research Support|Medline: Grant/Research Support|Merck: Grant/Research Support|Paratek: Grant/Research Support **Michael J. Satlin, MD**, AbbVie: IDMC member|Biomerieux: Grant/Research Support|Merck: Grant/Research Support|Shionogi: Advisor/Consultant|SNIPRBiome: Grant/Research Support **Martin Krsak, MD, MSc, FASAM**, AbbVie: Grant/Research Support|Melinta: Honoraria **W. Charles Huskins, MD, MSc**, ADMA Biologics: Advisor/Consultant|Bristol Myers Squibb: Stocks/Bonds|Pfizer: Advisor/Consultant|Pfizer: Stocks/Bonds|Zimmer Biomet: Stocks/Bonds **Robin Patel, MD**, Abbott Laboratories: Advisor/Consultant|Adaptive Phage Therapeutics: Grant/Research Support|Adaptive Phage Therapeutics: Mayo Clinic has a royalty-bearing know-how agreement and equity in Adaptive Phage Therapeutics.|BIOFIRE: Grant/Research Support|CARB-X: Advisor/Consultant|ContraFect: Grant/Research Support|Day Zero Diagnostics: Advisor/Consultant|HealthTrackRx: Advisor/Consultant|Mammoth Biosciences: Advisor/Consultant|Netflix: Advisor/Consultant|Oxford Nanopore Technologies: Advisor/Consultant|PhAST: Advisor/Consultant|See details: Patent on Bordetella pertussis/parapertussis PCR issued, a patent on a device/method for sonication with royalties paid by Samsung to Mayo Clinic|See details: continued, patent on an anti-biofilm substance issued|TenNor Therapeutics Limited: Grant/Research Support|Torus Biosystems: Advisor/Consultant|Trellis Bioscience, Inc.: Advisor/Consultant **Vance G. Fowler, MD, MHS**, Amphliphi Biosciences, Integrated Biotherapeutics; C3J, Armata, Valanbio; Akagera, Aridis, Roche, Astra Zeneca: Advisor/Consultant|Genentech, Regeneron, Deep Blue, Basilea, Janssen;: Grant/Research Support|Infectious Diseases Society of America: Honoraria|MedImmune, Allergan, Pfizer, Advanced Liquid Logics, Theravance, Novartis, Merck; Medical Biosurfaces; Locus; Affinergy; Contrafect; Karius;: Grant/Research Support|Novartis, Debiopharm, Genentech, Achaogen, Affinium, Medicines Co., MedImmune, Bayer, Basilea, Affinergy, Janssen, Contrafect, Regeneron, Destiny,: Advisor/Consultant|Sepsis diagnostic: Patent pending|UpToDate: Royalties|Valanbio and ArcBio: Stock Options **David van Duin, MD, PhD**, Entasis: Advisor/Consultant|Merck: Advisor/Consultant|Merck: Grant/Research Support|Pfizer: Advisor/Consultant|Pfizer: Honoraria|Qpex: Advisor/Consultant|Roche: Advisor/Consultant|Shionogi: Advisor/Consultant|Shionogi: Grant/Research Support|Union: Advisor/Consultant|Utility: Advisor/Consultant

